# Decomposition Behavior of Stereocomplex PLA Melt-Blown Fine Fiber Mats in Water and in Compost

**DOI:** 10.1007/s10924-022-02694-w

**Published:** 2022-11-28

**Authors:** Yahya Kara, Kolos Molnár

**Affiliations:** 1 Faculty of Mechanical Engineering, Department of Polymer Engineering, Budapest University of Technology and Economics, Műegyetem rkp. 3, Budapest, H-1111 Hungary; 2 MTA–BME Research Group for Composite Science and Technology, Műegyetem rkp. 3, Budapest, H- 1111 Hungary

**Keywords:** PLA, stereocomplexation, fiber mat, composting, hydrolysis, decomposition, melt blowing

## Abstract

This study introduces systematic and comparative investigations of various PLA fine fiber mats prepared by melt blowing. A series of PLLA and PDLA melt-blown fibers from various L and D enantiomers blends were produced. Their morphological, mechanical, and thermal properties were studied, and their decomposition in water and compost was investigated. It was found that the 1:1 ratio blend with stereocomplex crystals had an 80% lower average fiber diameter, 60% higher specific strength and better thermal stability than the PLLA and PDLA fiber mats. In the case of composting, the crystalline peak melting temperature, crystallinity, and thermogravimetric decomposition temperatures marginally decreased after 14 days. The high surface of the fine fiber mats played a crucial role in fast decomposition, as they entirely disintegrated in less than only 40 days. In the case of water, the homocrystalline domains were more susceptible to hydrolysis than the stereocomplex ones. All the PLA fiber mats underwent decomposition and extensive disintegration for 70 days in water. Hydrolysis reduced the amorphous and crystalline fraction of the fibers via surface and bulk erosion, while the decomposition of stereocomplex-crystalline-rich domains mainly exhibited surface erosion. Findings revealed that high porosity and the high surface area of PLA melt-blown fine fiber mats undergo fast decomposition in compost and in water.

**Graphical Abstract:**

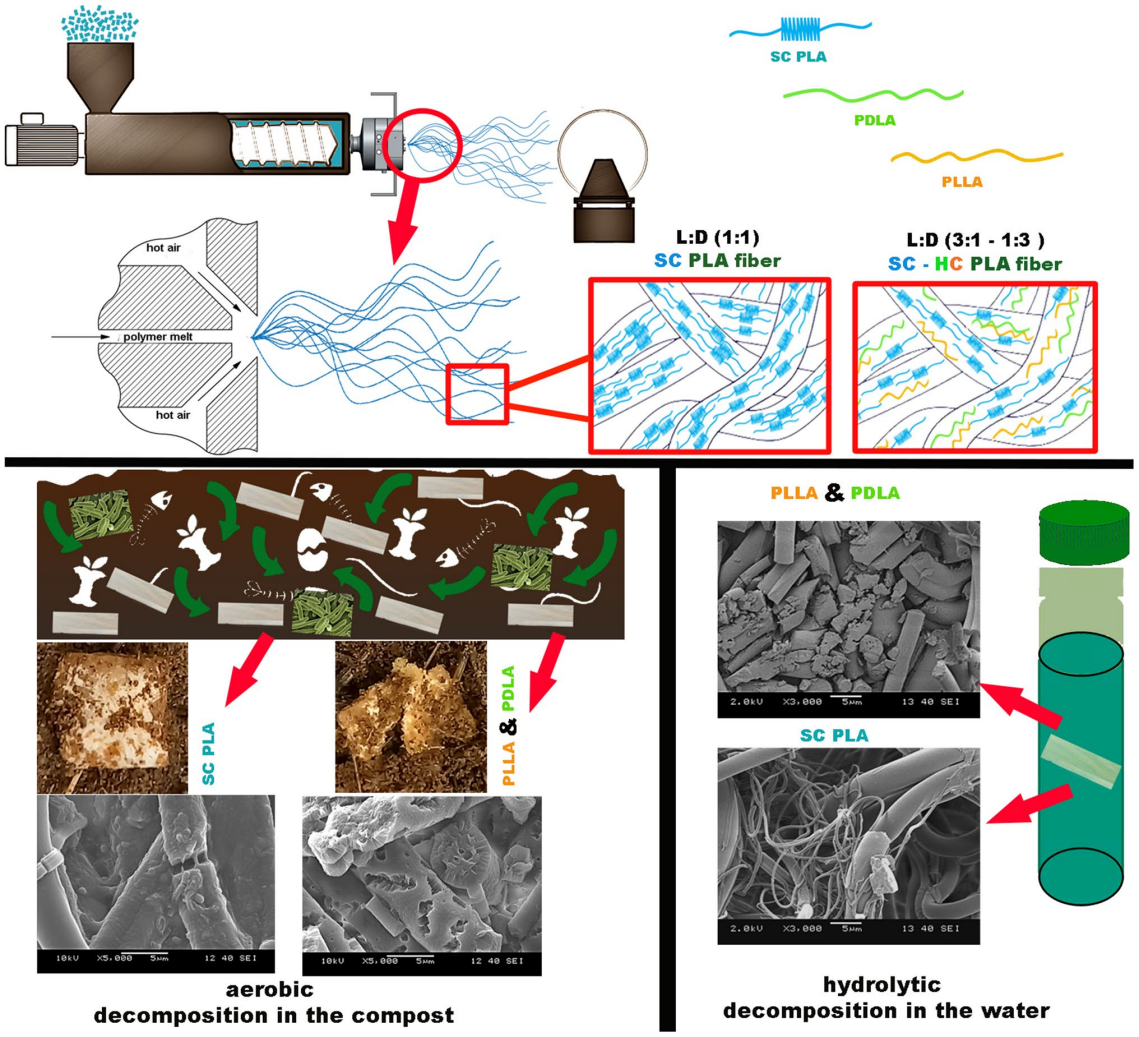

**Supplementary Information:**

The online version contains supplementary material available at 10.1007/s10924-022-02694-w.

## Introduction

Traditional, petroleum-based materials have been the norm in various domains and industrial applications due to their fair physical and mechanical properties and low cost. The tremendous and increasing amount of plastic waste has taken a toll on the environment [1]. Plastic waste is one of the most debated global environmental aspects humankind faces nowadays. In this regard, the non-biodegradability of most plastics is one of the most significant concerns. Moreover, a considerable proportion of plastic waste ends up in the ocean, especially in developing countries, where waste management is not adequately resolved [2, 3]. These concerns paved the road to the development of several aliphatic polyesters at the end of the 20th century. Besides the various daily life products and applications, biodegradable synthetic fine fibers are also gaining attention. Synthetic fibers have certain advantages over natural ones, e.g., smaller diameter with a smaller deviation, less moisture takeup, better performance, technically infinite length, etc. Melt blowing, comprising drawing of polymer melt by pressurized hot air, is one of the most prominent methods for making fine synthetic fibers in large volumes. MB fibers and related products are used in many applications, including but not limited to automotive, railway, aerospace, agriculture, geotextiles, industrial/military, medical/healthcare and construction [4–6]. Among them, demand for personal protective equipment (PPE) made of MB fibers has soared to unprecedented levels due to the COVID-19 pandemic. Reports indicate that only in 2020, over 50 billion face masks were produced, while it is estimated that 1.6 billion of these masks ended up in oceans [7]. The automotive nonwoven market worldwide was rated around 10 billion USD in 2018, while it is expected to extend to 14.2 billion USD by 2026 [8]. Yet, most of these nonwovens are not entirely environmentally friendly since they are made of non-biodegradable polymers, such as PP.

In this regard, polylactic acid (PLA), poly(butylene succinate), (PBS), poly(ε-caprolactone (PCL), poly(3-hydroxy alkanoates) (PHA), poly(hydroxybutyrate) (PHB) and their derivatives, so-called green polymers, are introduced in the market [9–12]. PLA is a promising alternative used extensively in biopolymer production, and the market has grown in the last three decades. The current global production is estimated to be 0.5 million tonnes per year, which contributes to the total bioplastics production by 18–20% [13]. PLA is a compostable and bio-based polyester, first discovered in 1932 by Carothers, and after further refinements, Dupont patented Carother’s process [14]. Although PLA has fairly good mechanical properties (e.g., high tensile strength and elastic modulus), some of the inherent properties of PLA are hindering its spread in the industry (e.g., low elongation at break, poor thermal stability, hydrophilicity, high moisture and oxygen permeability, low impact resistance) [15, 16].

In order to surpass these disadvantages, stereocomplex (SC) crystallization is a promising and reliable way of improving PLA’s thermal and mechanical properties. Back in 1987, Ikada et al. [17] first reported the SC formation in poly(l-lactide) (PLLA)/poly(D-lactide) (PDLA) enantiomers. As opposed to HCs, the hydrogen bonding and dipole-dipole interactions between the PDLA and PLLA enantiomers provide tightly-packed chain conformations of SC crystallites [18]. As a result, the SC PLA exhibits a peak crystalline melting temperature about 50 °C higher than that of PLA containing homocrystallites (HCs). Furthermore, the PLLA or PDLA enantiomers with HC inherently have lower mechanical properties than the SC crystals; thus, the SC crystals are favorable for advancing applications of the PLA biopolymers.

The crystalline structure also plays a crucial role in the decomposition of PLA; however, the results presented in the literature are contradictory. Tsuji [19] found that the degradation rate of SC PLA films immersed in a phosphate buffer solution was low, and the SC domains were more hydrolytically stable than the HC ones. On the other hand, Serizawa et al. [20] reported that the alkaline hydrolysis of SC PLA was faster than that of HC, and it was similar to the behavior of amorphous PLA. Kawai et al. [21] studied the SC PLA film. They blended PLLA and PDLA in a ratio of 50:50 and investigated the depolymerization via hydrolysis in the cutinase-like enzyme (CLE). Their findings showed that the SC PLA film was only slightly degraded, mainly at the amorphous interface, and the remaining excess PDLA chains formed homo crystals in α form. Gil-Castell et al. [22] developed a PLA self-reinforced composite (PLA-SRC) made of a PLA spunbond (SB) nonwoven and a PLA extruded film, and they investigated the PLA SRC’s composting, hydrolysis, and thermal degradation behavior in parallel. They found that the higher crystallinity of the SRCs enhanced thermal, composting, and hydrolytic degradation. However, the composites subjected to thermal and hydrolytic degradation for more than 70 days showed a decrease in the degree of crystallinity, indicating degradation crossed over crystalline regions after a severe deterioration of the amorphous domains. They reported no change in a macroscopic structure subject to thermal degradation (at 58 °C) for 147 days. On the other hand, hydrolysis (bidistilled water) promoted erosion and severe decomposition over 147 days. Their findings showed that the composites entirely degraded over 35 days under composting conditions.

Many efforts have been made to unfold the biodegradation and decomposition of PLA and its derivatives, mainly for films or injection molded products. In this context, Moreno Nieto et al. [23] reported the design of a 3D-printed oceanographic buoy made from PLA and polyethylene terephthalate glycol (PETG). In their study, the degradation and water absorption properties of 3D-printed PETG and PLA material specimens were evaluated. They also detailed an approach to develop a biopolymer-derived end-product with weight reduction and fairly good hydrolysis resistance. Palai et al. [24] investigated the composting of blown films made of PLA and its blend with thermoplasticized starch (TPS) and poly(butylene succinate-co-adipate) (PBSA) for packaging applications. Their results showed that PLA/TPS film showed an optimum degradation rate with a half-life of 103 days, followed by PLA/PBSA and pristine PLA films having a half-life of 559 days and 835 days, respectively. The main idea behind these efforts is to show carbon dioxide neutrality through biodegradation during the operation and after their service life contributing polymer waste disposal problem. Up to date, limited attention has been devoted to the degradation and decomposition of biopolymer-based nano- and micro fibrous structures. Fiber mats and related products global market rated $44.37 billion in 2017, and it is expected to reach a market value of $98.78 billion by 2026 [25]. Fiber mats and related products are potential risks for environmental pollution; therefore, it is necessary to understand their decomposition characteristics, recycling capabilities, etc. In this regard, Arrieta et al. [26] studied the disintegration of the oligomeric lactic acid (OLA) plasticized PLA:PHB (1:3) blend electrospun fibers under lab-scale composting conditions. They found that OLA plasticizer speeds up the disintegration, while PLA:PHB fiber mats entirely disintegrated under composting conditions in less than 60 days. Recently, Wang et al. [27] developed a PLA single-use face mask via electrospinning (with an average fiber diameter of 37±4 nm and tested its composting (soil from China, Henan) biodegradability. Their results showed that the PLA nanofiber filter had 99.996% particle capturing efficiency and low air resistance of 104 Pa, while the filter media fully disintegrated during outdoor soil burial after 150 days. Another study conducted by Choi et al. [28] reported a hierarchical filter media made of chitosan nanowhiskers coated poly(butylene succinate) (PBS) electrospun nanofibers. The developed filter media showed a 98% efficiency (PM_2.5_) and low-pressure drop (59 Pa), while it completely decomposed in the composting soil within a month. Dharmalingam et al. [29] investigated degradation in the soil of commercial PLA, PHA, and PLA/PHA blend nonwovens made by melt blowing and spun bonding for agricultural mulch applications. They concluded that the melt-blown (MB) PLA/PHA nonwoven had a higher decomposition than that of SB nonwovens. They also reported that the average diameter of the MB fibers (15.3±0.6 μm) was slightly thinner than that of the SB fibers (18.2±0.6 μm), while blending almost doubled the average fiber diameter.

Compared to the homocrystalline PLA, the PLA SC is far less investigated. In this regard, we produced PLA SC via melt stereocomplexation using a twin-screw extruder, followed by generating fine fiber mats via melt blowing. We conducted a systematic and comparative analysis of PLA fine fibers’ hydrolytic and composting behavior, for which we used differential scanning calorimetry (DSC), thermogravimetric analysis (TGA), scanning electron microscopy (SEM), and visual/optical inspections. We tested and summarized the stereocomplex, homo crystalline, and blend fiber mats’ decomposition behavior, accordingly.

## Materials and Methods

### Materials

PDLA homopolymer (Total Carbion, Luminy® D070, MFI: >100 g/10 min at 190 °C, 2.16 kg) and PLLA homopolymer (Total Carbion, Luminy® L105, MFI: 70 g/10 min at 190 °C, 2.16 kg) resins were used for the preparation of fine fiber mats via melt blowing. All resins used in this study were dried in a WGL 45B oven (Huanghua Faithful Instrument, China) at 100 °C for 4 h.

### Producing Stereocomplex PLA Fiber

PLLA and PDLA blends in a weight ratio of 0:1 (PLLA), 3:1 (3D1L), 1:1 (1D1L), 1:3 (1D3L) and 1:0 (PDLA) were prepared by dry mixing. The PLLA and PDLA were then blended using a twin counter-rotating screw extruder (Labtech LE 25–30/C, Thailand). The extruder had two pieces of 26 mm diameter, 44 L/D ratio screws. The related extrusion parameters are given in Table 1. Continuous filaments through the extruder die with two circular orifices were produced and then granulated by an LZ-120/VS type granulator (Labtech, Thailand).

**Table 1:** Extrusion compounding parameters

Twin-screw extruder zones and corresponding extrusion temperatures				Rotation speed	
1–2	3–5	6–8	9–10 (die)	screw	feeder
220 °C	225 °C	230 °C	235 °C	60 rpm	15 rpm

**Fig. 1:**
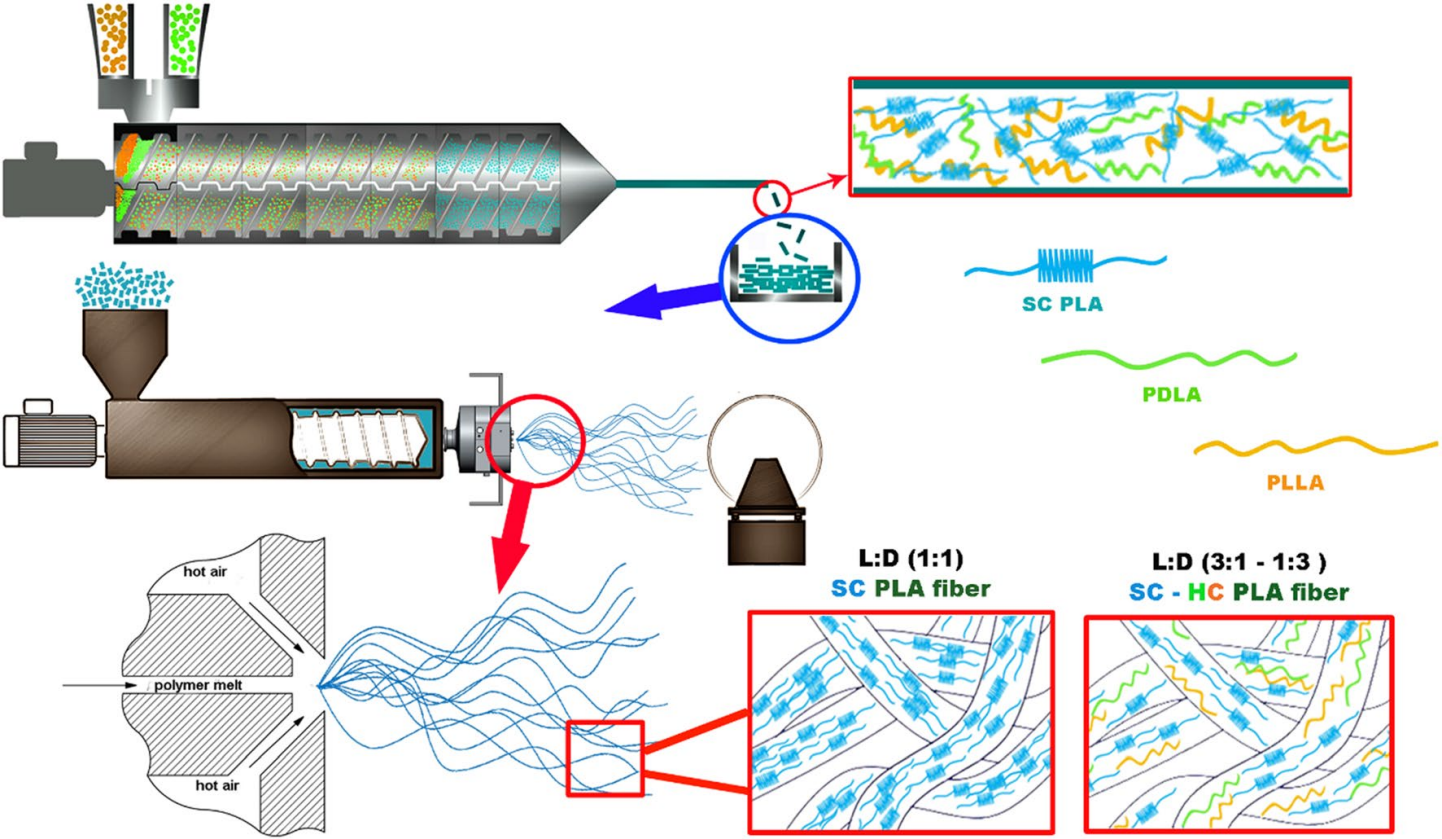
Schematic of the melt stereocomplexation and related MB PLA fiber production via melt blowing

A custom laboratory unit (Figure S 1) was used to make the melt-blown fibers. A custom dual-slot melt blowing die was mounted to a LE8-24 C type single-screw laboratory extruder (LabTech, Thailand). The extruder had a single screw with 8 mm diameter and 24 L/D ratio screws. The die operates with 40 fiber-forming capillaries, 330 μm diameter each, arranged in a single row. The die and extruder temperatures were set to 240 °C. The air temperature and pressure were set at 250 °C and 2 bar, respectively. The extruder screw speed was set at 1 rpm. The die-to-collector distance (DCD) was set to 200 mm. A drum with a diameter of 160 mm was used as the collector. The circumferential velocity of the collector was set at 28 m/min. The sample collection time was 10 min for all the compositions. The melt stereocomplexation and melt blowing fiber generation are shown in Fig. 1.

### Characterization by Differential Scanning Calorimetry (DSC) and Thermogravimetric Analysis (TGA)

The thermal properties of the MB fibers were determined by DSC (Q2000, TA Instruments, USA) device. The tests were performed on samples with a mass of ~ 3–7 mg in an inert atmosphere (N_2_; 50 ml/min purge flow rate) with a heating and cooling rate (heat ramp) of 5 °C/min covering a temperature range of 0 to 250 °C. The fibers’ degree of crystallinity (DoC) was determined based on Eq. 1. For the heat of fusion of the 100% crystalline (${\varDelta H}_{m}^{0}$) stereocomplex (SC) PLA 147 J/g [30], while 93 J/g [31] for the homocrystalline (HC)

PLA was used in the calculations (1):

1 $$\chi \, = \,\frac{{\Delta H_{m} \, - \,\Delta H_{{CC}} }}{{\Delta H_{m}^{0} }}\, \times \,100\,[\% ]$$

where, $\Delta H_{m}$and $\Delta H_{{cc}}$ are the experimental enthalpy of the heat of fusion and the experimental enthalpy of cold crystallization obtained by the DSC scans, respectively.

To characterize the fiber mat’s thermal stability, TGA was performed using a TGA Q500 (TA Instruments, USA) thermogravimetric analyzer between 50 and 600 °C. The tests were carried out on samples with a mass of ~ 5 mg at a heating rate of 10 °C/min under nitrogen purge flow (60 ml/min).

## Tensile Tests

PLA MB fiber mat samples were cut in 40 mm x 10 mm size rectangles. The tensile properties of the fiber mats were tested at room temperature with a Zwick Z005 (Zwick, Germany) type universal tensile tester equipped with a 20 N maximum capacity load cell. The fiber mat length and width were measured using a caliper gauge (Fowler Promax) with a precision of 0.01 mm, while their thickness was measured using a micrometer (Louis Schopper Leipzig, Germany) with an accuracy of 0.01 mm. MB fiber mats were weighed using a Sartorius Quintix 125D-1CEU (Sartorius, Germany) semi-micro scale. The fiber mat’s cross-section area was calculated by using Eq. 2.

2 $$A = \frac{{{\text{m}}_{{fm}} }}{{l_{{fm}} \rho _{{PLA}} }}$$

where, ${\text{m}}_{fm}$ is the fiber mat mass, ${l}_{fm}$ is the length of the fiber mat specimen, and ${\rho }_{PLA}$ is the bulk density of PLA (1,240 kg/m^3^).

### Scanning Electron Microscopy (SEM)

The morphology of the fibers was observed by using scanning electron microscopy (SEM; JEOL 6380 LA, Japan). MB fiber mats were pasted carefully onto metallic studs with double-sided conductive tape. The surface of the fiber mat samples was finely coated using a JEOL JFC-1200 (Jeol Ltd., Japan) fine coater with gold (Au) to avoid their charging. We measured 100 fibers for each sample to analyze the fiber diameter distributions. ImageJ 1.51k software was used for this measurement.

### Composting Study

Composting conditions were set and ensured according to the ISO-20,200 standard [32]. The reactor included a mixture of 40% sawdust, 30% rabbit feed, 10% ripe compost, 10% corn starch, 5% sugar, 4% corn oil, and 1% urea. Bidistilled water was added in a 45:55 ratio into the mixture. Bidistilled water was added periodically to the reaction container to maintain the relative humidity in the compost medium according to the same standard. Fiber mats were cut (30 × 30 mm^2^) and buried a few cm deep in plastic reactors containing the compost medium. Reactors were then introduced in a climate chamber (Climacell 111, MMM Group, Germany) at 58 °C. Fibers were recovered from the disintegration container at different times (7, 14, 21, 28 and 35 days) and dried in a vacuum drying chamber (VD 53, Binder GmbH, Germany) at 30 °C under vacuum for 72 h. Composting and disintegration were photographed for all samples periodically.

### Hydrolytic Degradation Study

PLA MB fiber mat’s hydrolytic degradation tests were conducted in bidistilled water at 58 °C. Since this temperature was given for composting and disintegration tests according to the ISO standard, we adopted this temperature for the hydrolytic degradation studies as well. Several rectangular pieces of fiber mats of each sample type were placed in glass vessels containing bidistilled water. The mass amount of water was fixed in the ratio 500:1 with the mass of the fiber mat sample. In the first 4 weeks, each vessel was emptied using a fine filter paper every week. From the 5th week, each vessel was emptied fortnightly until the 10th week. Fresh bidistilled water was then added to the vessel containing the sample, and the vessel was put again in water at 58 °C. The bidistilled water amount was kept the same. Based on the literature data, we applied this routine of replacing the bidistilled water to prevent the influence of the pH change caused by hydrolysis [33–35]. The samples were removed from the hydrolytic medium: one sample per week from week 0 to 4 and one sample fortnightly between week 4 and 10. The extracted samples were dried in a drying chamber (VD 53, Binder GmbH, Germany) at 30 °C under vacuum for 72 h.

## Results and Discussion

### Generating HC and SC PLA Melt-Blown Fiber Mats and Their Characteristics

**Fig. 2:**
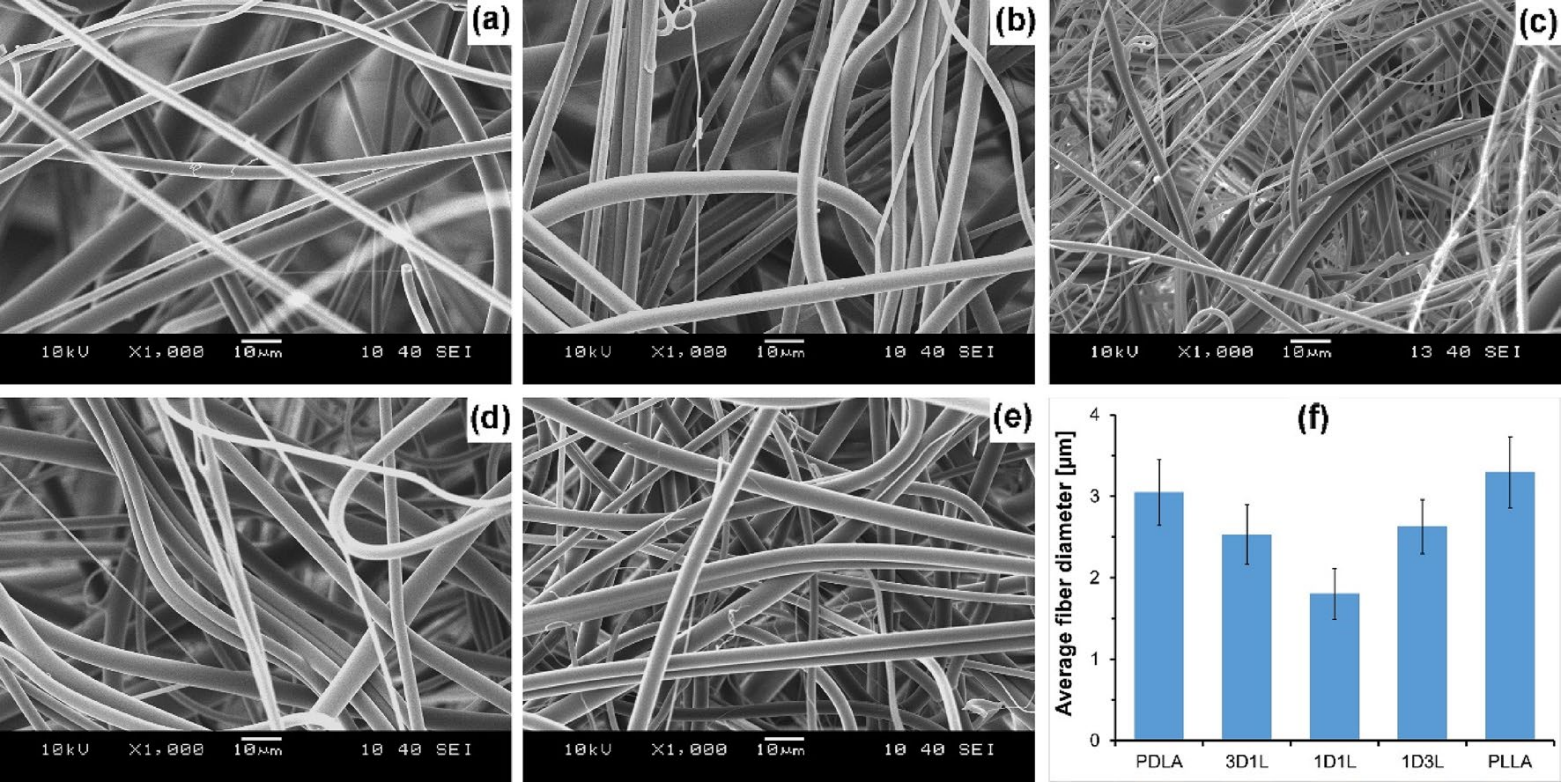
MB fiber mats SEM images; **a** PDLA, **b** 3D1L, **c** 1D1L, **d** 1D3L, **e** PLLA and **f** average fiber diameters of the fiber mats

PDLA, 3D1L, 1D1L, 1D3L and PLLA PLA MB fiber mats were generated with average diameters ranging from 1.8 to 3.3 μm via melt blowing. The fiber mat is formed continuously, and the polymer melt is drawn by pressurized hot air without controlled stretching. The SEM images and fiber diameters are shown in Fig. 2, while fiber diameter distributions are shown in Figure S 2.

Results showed that 1D1L MB fiber mats had the smallest average fiber diameter, 1.8±0.3 μm, among the other samples, while the PLLA fiber mat had the largest average fiber diameter, 3.3±0.4 μm. We observed that the 1D1L fiber mat had nearly 70 to 80% thinner fiber diameter, while 3D1L and 1D3L had 20 to 30% compared to the PDLA and PLLA fiber mats. The stereocomplexation resulted in strong and physically crosslinked crystalline domains, as shown in Fig. 1. As a result, the molecular chains are confined to the network structure in the blended melt. This phenomenon might cause more pronounced local stresses in the network system; hence, larger deformation might occur between strong SC crystalline domains [36]. This is similar to “Tug of War” proposed by Deng et al. [37]. They reported that PP/PS blend MB fiber diameter decreased with increased PS content due to the continuously fluctuated melt viscosity of the blend. These findings imply that high SC content (i.e., 1D1L) led to a formation of hard domains (i.e., confined, network structure) along the SC molecular chain, caused high local stress during the fiber formation and resulted in a significant decrease in the fiber diameter.

**Fig. 3:**
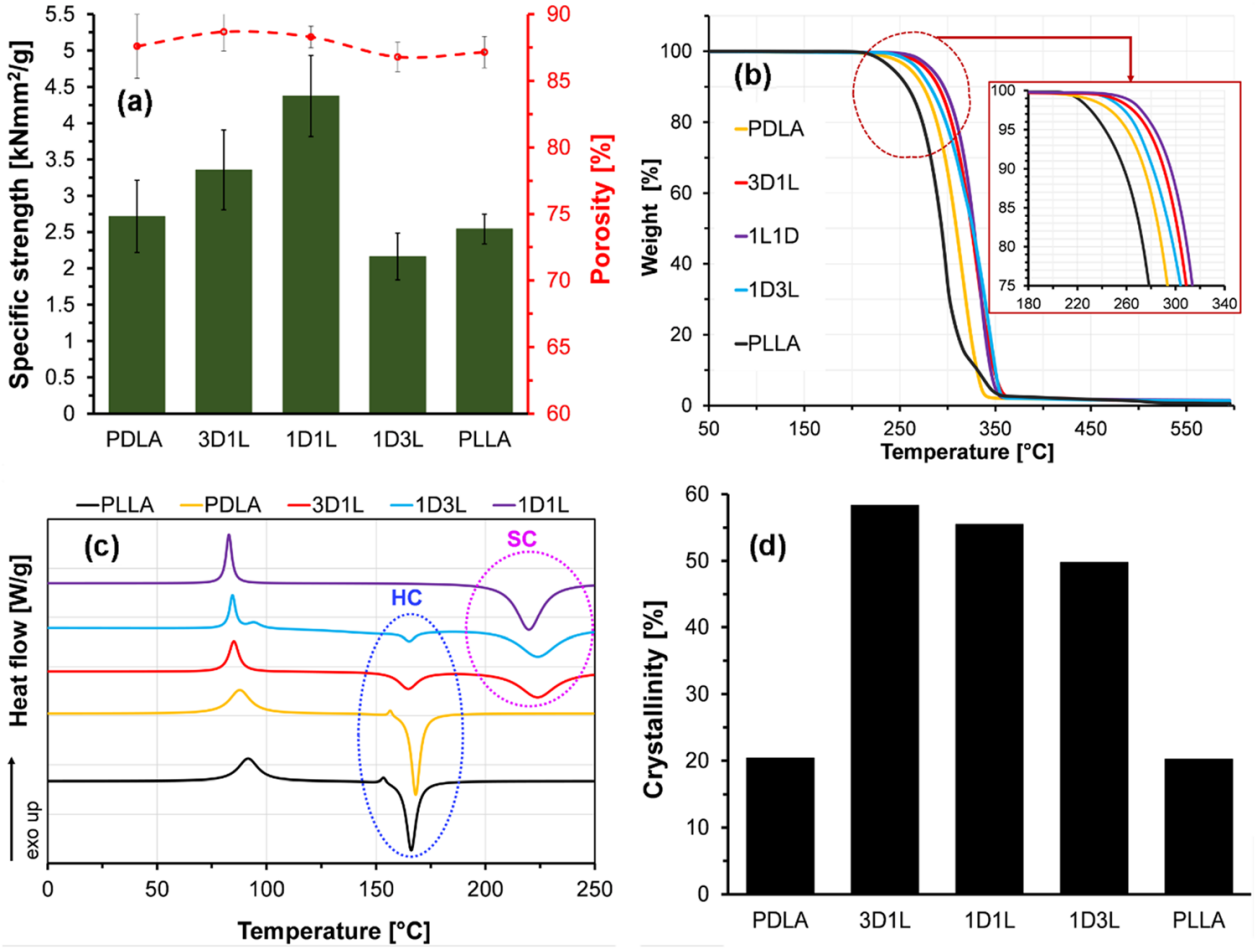
Characteristics of the SC and HC PLA MB fiber mats; **a** specific strength and porosity, **b** TGA curves, **c** 1st heating thermograms and **d** crystallinity variation for the neat and blend fibers

The PLLA and PDLA fiber mats had nearly the same specific stiffness, while that of the fiber mats made of 3D1L and 1D1L blends were higher, as shown in Fig. 3 (a). However, the specific strength of the 1D3L fiber mat slightly decreased due to melt-blowing instabilities (Figure S3). Such instabilities and defects are unfavorable for the melt-blowing process and result in uneven fiber morphology (i.e., droplets, fiber shots, flies) [38]. The 1D3L fiber mats had fiber shots and droplets, which weakened the fiber mat’s strength. On the other hand, all the PLA fiber mat’s porosity did not show a significant change (Fig. 3 (a)).

TGA results revealed that the degradation temperature of the SC PLA fibers shifted to a higher temperature due to the presence of the SC crystallites, as shown in Fig. 3 (b). The PDLA fiber mat exhibited slightly higher thermal stability than the PLLA fiber. This is attributed to the formation of thinner PDLA fibers compared to the PLLA fibers. The low PDLA viscosity resulted in larger attenuation of the polymeric jets, which translated to enhanced molecular orientation and thermal stability [39, 40]. The 1D1L fiber mat decomposition temperatures, at which 95% and 50% of the mass remained was nearly 40 °C higher than those of PDLA and PLLA fiber mats. Results showed that the denser chain packing and the stronger intermolecular stereocomplexation exhibited enhanced thermal stability and heat resistance.

DSC findings showed that SC formed for the 1D1L fibers (1:1), while both HC and SC took place in the blends with asymmetric compositions (1:3 and 3:1), as shown in Fig. 3 (c). In this regard, the D and L enantiomers DSC 1st heating melting peak was observed between 165 and 175 °C (Fig. 3 (c)), which is attributed to the melting of HC domains. The HCs are composed of either PLLA or PDLA chains. On the other hand, SC crystallites are formed when PLLA and PDLA molecules are packed side by side [41]. The excess amount of PDLA or PLLA enantiomers remained without participating in the SC formation; therefore, some HC domains were observed at the DSC thermograms as an endothermic peak around 170 °C (Figs. 1 and 3 (c)). The fiber mat’s characteristics primarily depend on the degree of stereocomplexation [42]. 1D3L, 1D1L and 3D1L fiber mats degree of SC crystallinity were 33.8, 56.7, and 55.5%, respectively. On the other hand, 1D3L and 3D1L fiber mats SC and HC total degree of crystallinity was found to be 49.7 and 58.3%, respectively, as shown in Fig. 3 (d). These findings indicated that the degree of crystallinity of SC crystals increases with increasing D lactic content. The degree of crystallinity of the SC-PLA fiber mat was up to 56% when the PDLA content was 75 wt%. Results implied that increasing D content restrained the crystallization of HC crystals due to the strong SC crystalline network. It was found that the HC degree of crystallinity was decreased with increasing D content while the SC crystalline formation and the fiber mat’s degree of crystallinity were increased. These findings are in-line with previous reports that indicate increasing D content increases the degree of crystallinity in the PLLA/PDLA blends [39, 43–47].

Results showed that melt blowing resulted in a large extent of SC crystal formation. The dominant SC formation is associated with a higher intrinsic orderliness, restricted by the fiber boundary. Besides, the high shearing force caused by high-velocity air led to improved interaction between the PLLA and PDLA chains, which resulted in the formation of SC by suppressing the formation of HCs [48]. These results confirmed that melt blowing improves the fibers’ thermal stability and specific strength by enhancing the SC content, as shown in Fig. 3 and Figure S4.

### Evaluating Degradation and Disintegration of MB PLA fiber Mats Under Composting Conditions

**Fig. 4:**
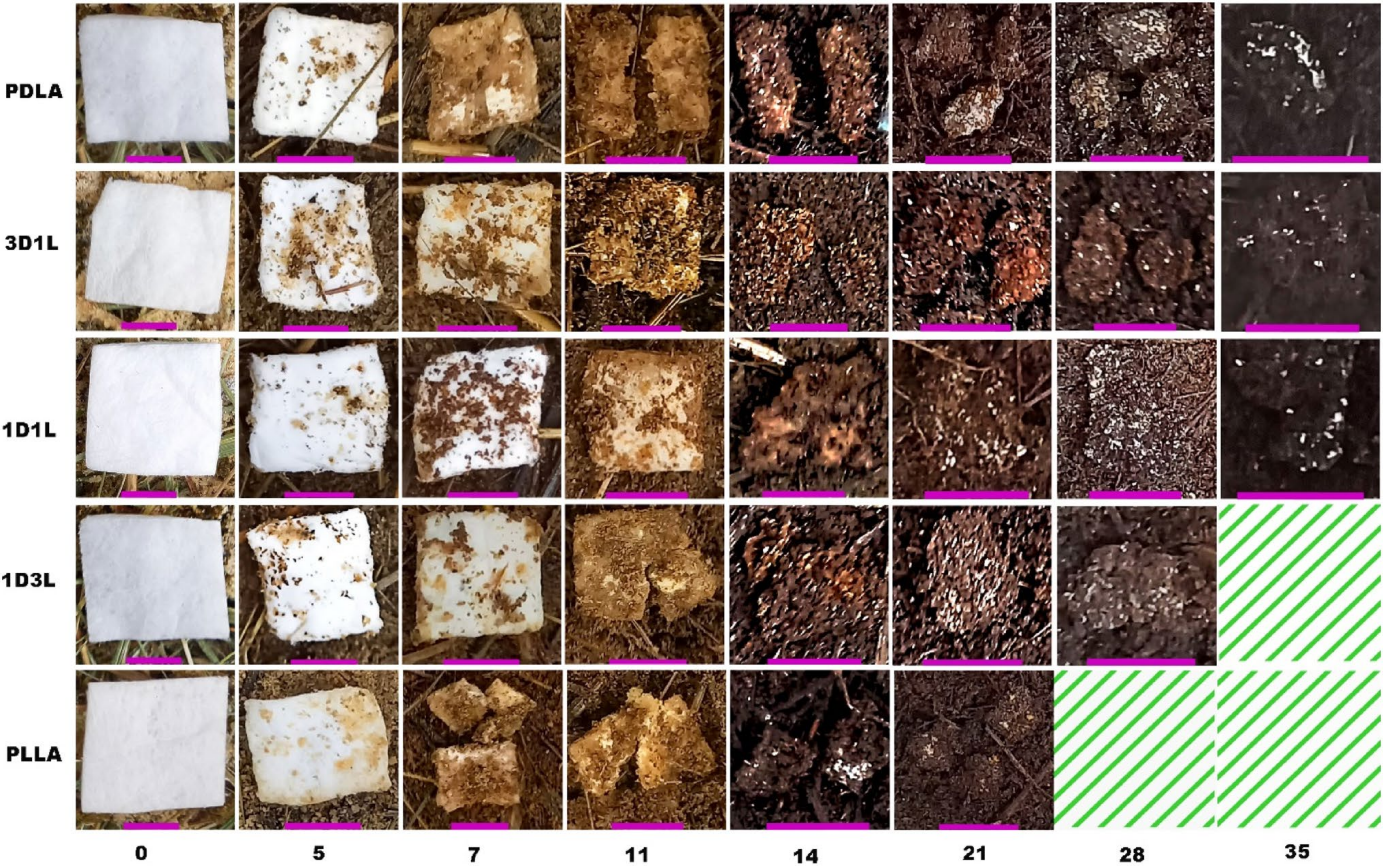
Optical images of MB fiber mats at different decomposition times (purple color bar represents 10 mm)

We evaluated the composted PLA fiber mats’ decomposition behavior by SEM, DSC, and DTG. The fiber mat decomposition was also analyzed via visual inspection from the recovered pieces, as shown in Fig. 4. The PLLA fiber mat showed the lowest resistance against decomposition and exhibited fiber ruptures and signs of erosion on the fiber surfaces after 7 days of composting, while PDLA and 1D3L samples followed similar behavior after day 11. By day 14, the degree of erosion became severe, and all the fiber mats presented an intensive deposition of composting matter on the surface. It seems that the composting matter fiercely bonded to the fibers, thus causing high interaction of microorganisms from day 14.

**Fig. 5:**
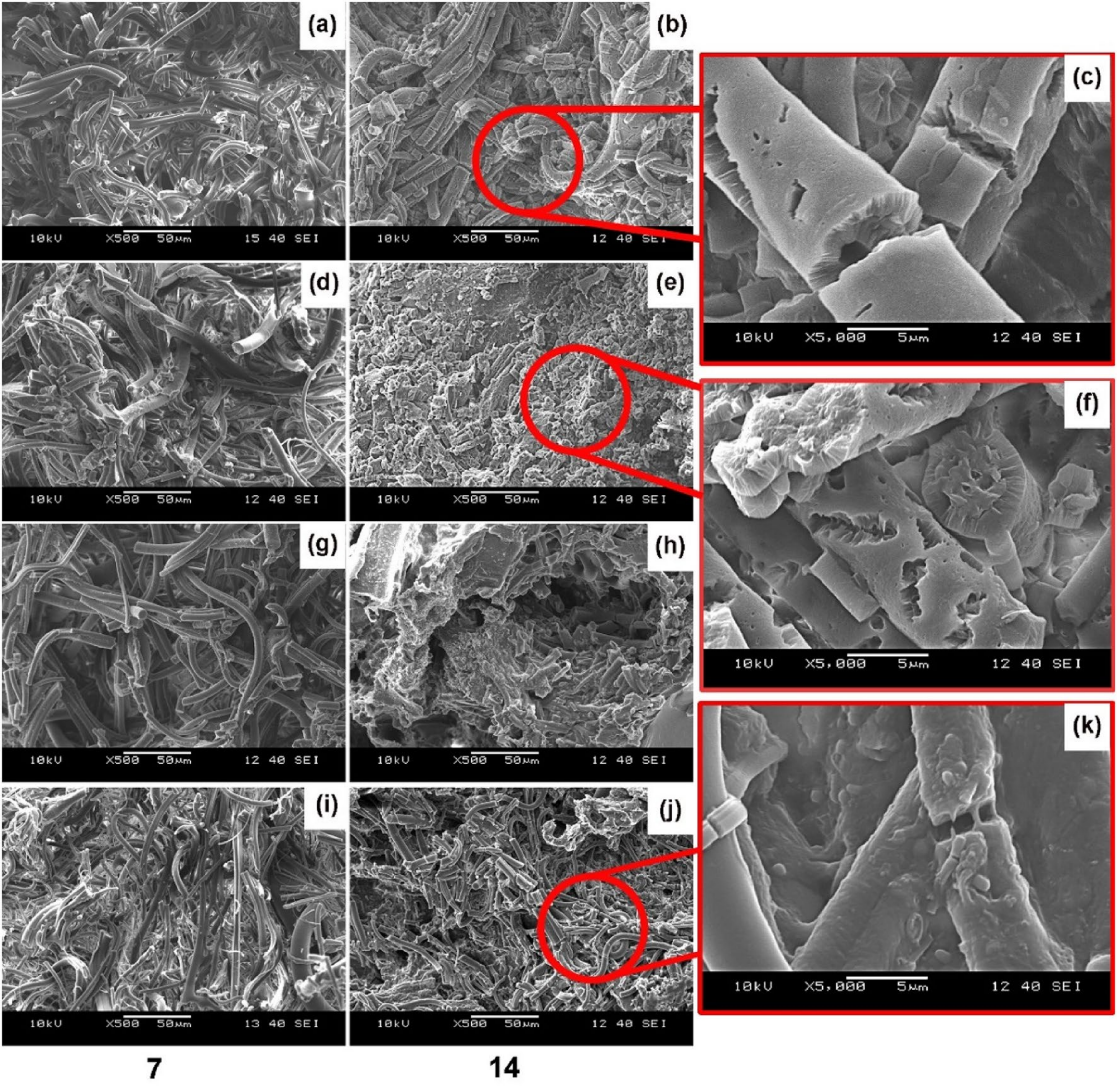
SEM images of MB fiber mats after 7 and 14 days of disintegration & composting times; **a**–**c** PLLA, **d**–**f** PDLA, **g**–**h** 1D3L and **i**–**k** 1D1L

**Fig. 6:**
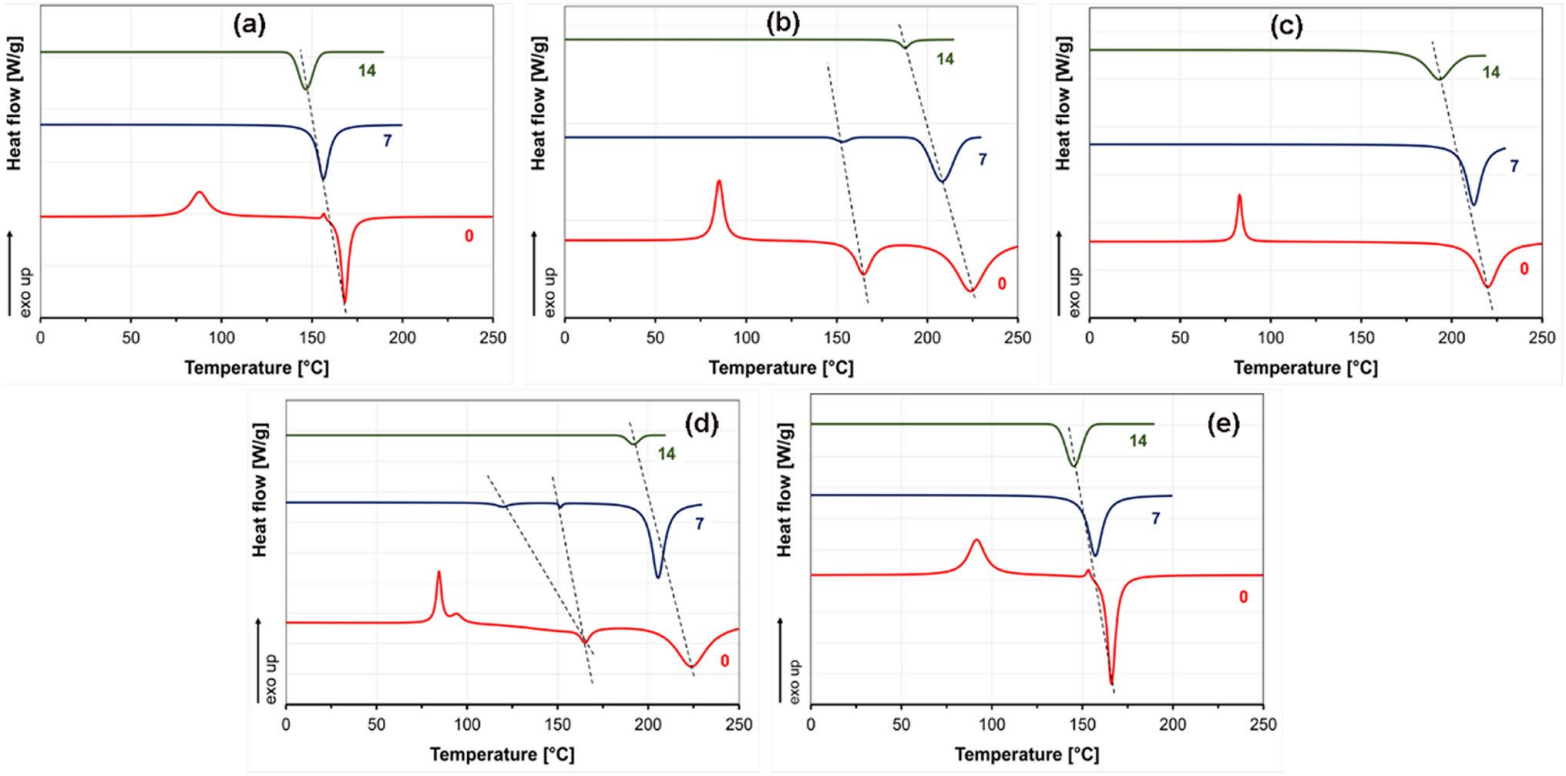
DSC 1st heating thermograms of the fiber mats; **a** PDLA, **b** 3D1L, **c** 1D1L, **d** 1D3L and **e** PLLA at different decomposition times

Results showed that the PLLA fiber mat had the fastest composting disintegration, while the 1D3L and PDLA fiber mats followed this. The 1D1L fibers showed higher resistance against disintegration and degradation. The test sample’s thickness, physical form (e.g., fiber, film, etc.) and structure (i.e., crystallinity) play a crucial role in the decomposition rate [26, 49, 50]. SEM investigations suggested that PLLA and PDLA fiber mats had an extensive surface to bulk erosion and fiber rupture, as shown in Fig. 5. The composting-induced structural evolution illustrates extensive bulk erosion after 14 days, as shown in Fig. 5 (c and f). On the contrary, the 1D1L fiber mat showed fiber ruptures (i.e., brittle fracture) and a trace of surface erosion (Fig. 6k). The 3D1L and 1D3L fiber mats exhibited a similar degradation mechanism as the 1D1L SC fiber mat. SEM findings suggest that SC content somewhat slowed down the bulk erosion mechanism. However, all the PLA fiber mats were fully disintegrated under composting conditions in less than 40 days, indicating a high surface area of fibers might boost a faster degradability in compost regardless of the molecular structure [51, 52].

Composting behavior via TGA and DSC was only evaluated until day 14 because, from day 21, the composting matter content on the fibers hampered the DSC and TGA analysis. The DSC data on day 21 showed a baseline only, which is why these data were not included in the DSC and DTG data evaluation. In addition, this phenomenon also reveals how fast fibers placed in compost decompose. The fast decomposition might be related to the thin fibers with high surface area and high porosity [51, 52]. Results showed that the crystalline melting temperature of the 3D1L and 1D3L fibers dramatically decreases with increasing decomposition time, as presented in Fig. 6 and Table S 1. These results suggest that the composted PLA fiber mats underwent continuous deterioration in the amorphous and crystalline domains regardless of the crystalline structure.

**Fig. 7:**
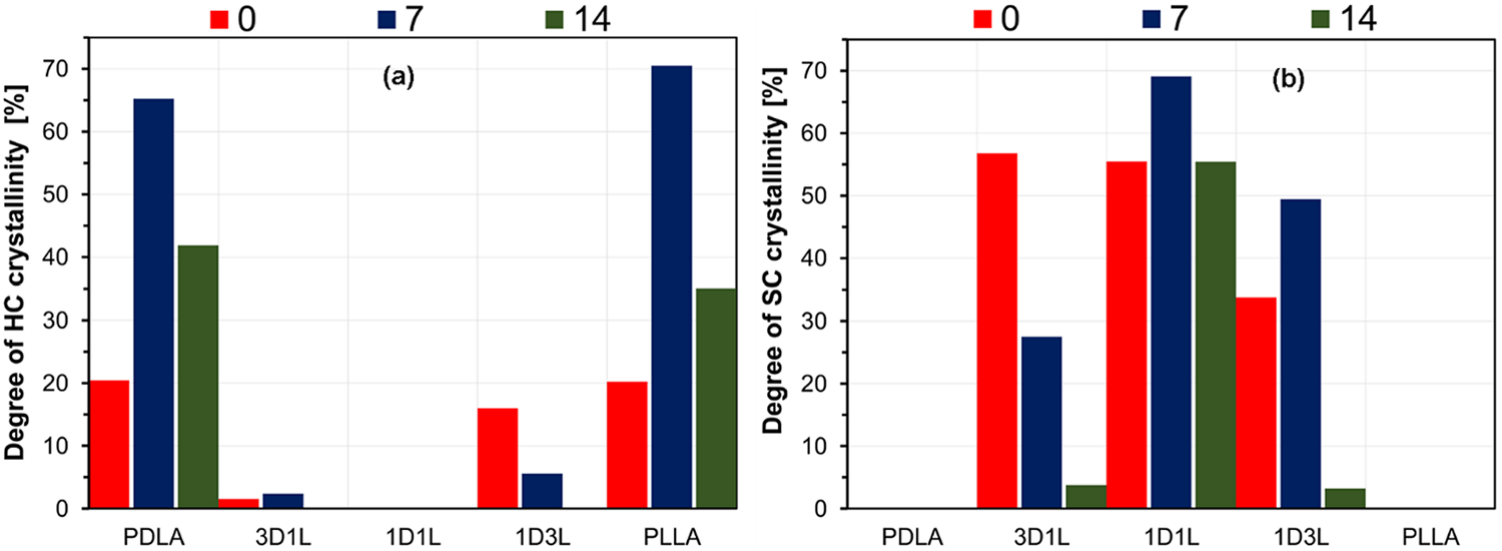
Degree of crystallinity of **a** homocrystalline (HC) and **b** stereocomplex (SC) crystals after different decomposition times, obtained from the DSC 1st heating

Results implied that fiber mat crystallinities increased after 7 days in the composting test. Even though the fiber-making process yields a highly oriented crystalline structure, PLLA and PDLA fibers had a very low degree of crystallinity after the melt blowing (day 0). However, the composting induced a higher degree of crystallinity over the 14 days decomposition time for PLLA and PDLA fiber mats, as shown in Fig. 7 and Table S 2. This might be attributed to the intrinsic water-absorbing capacity of PLA [53]. The PLA’s amorphous domains tend to take up water, which initiates hydrolytic degradation mechanisms before a possible degradation begins in the crystalline regions. This yields an increased ability to reorganize for molecular chains and the space available in the domain. As a result, polymeric material could exhibit increasing crystallinity alongside rigidity and fragility [54, 55]. On day 7, the PLLA and PDLA fiber mat crystallinities were raised to 65 and 70%, respectively. Then, the degree of crystallinity significantly decreased to 35–40% after day 14, which might be related to the decomposition of the crystalline fraction [22]. We observed similar characteristics for the 1D1L fiber mat after 7 days of composting. On the other hand, 3D1L and 1D3L fibers showed the opposite behavior with a continuous decrease in the degree of crystallinity over the 14 days decomposition time. The 3D1L and 1D3L fibers showed no HC content in the day 14 DSC thermograms. This finding implies that composting started at HC domains followed by SC domains in the 3D1L and 1D3L fibers. On day 14, the 3D1L and 1D3L fibers’ degree of crystallinity reduced drastically, suggesting a fast decomposition rate under composting conditions. The 1D1L fibers degree of crystallinity first increased to nearly 70% after 7 days, then reverted to 55%.

**Fig. 8:**
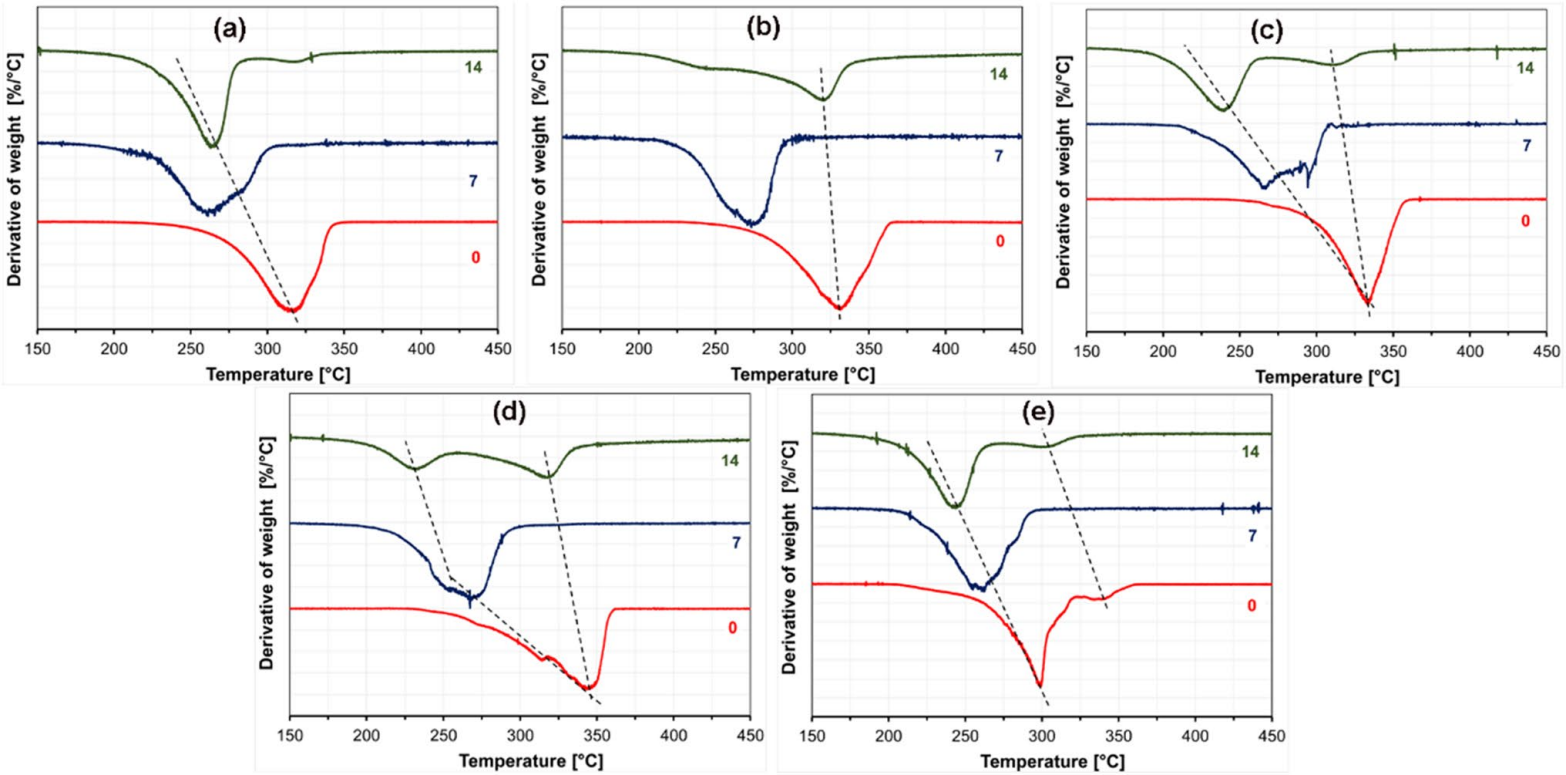
DTG curves of the fiber mats; **a** PDLA, **b** 3D1L, **c** 1D1L, **d** 1D3L and **e** PLLA at different decomposition times

DTG curves suggest that fibers decomposed gradually during the composting, as shown in Fig. 8. The results are in good agreement with the degree of crystallinity and melting temperature decrease. The initial mass loss and the final decomposition temperature dramatically shifted to lower temperatures, indicating reduced molecular weight. Results showed that the SC and HC fiber’s maximum mass loss rate temperature decreased significantly after 7 days of composting. A cliff fall in initial mass loss and maximum mass loss rate temperature was observed after 7 days, attributed to fast hydrolytic degradation in the composting medium. However, the final decomposition temperature did not change significantly for all the PLA fiber mats (~ 340 °C) on day 14. The decomposition temperature became broader with increasing decomposition time, evidencing the crystalline phase transitions and subsequent degradation.

### Analyzing the MB PLA Fiber Mats Decomposition on the Hydrolytic Medium

**Fig. 9:**
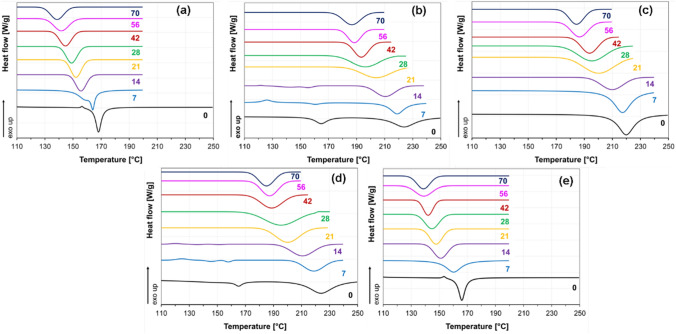
DSC 1st heating thermograms of the fiber mats; **a** PDLA, **b** 3D1L, **c** 1D1L, **d** 1D3L, and **e** PLLA at different decomposition times

The DSC results revealed that the peak melting temperatures shifted to lower temperatures (Fig. 9). The detailed DSC data are given in Table S 3 and Table S 4. After 21 days of immersion, HC crystal melting endotherms and cold crystallization exotherms disappeared for the 3D1L and 1D3L fibers. After 14 days in water, cold crystallization exotherms also disappeared for all the PLLA and PDLA samples. These findings imply that all PLA fibers were undergone hydrolysis. For the HC domains of the PLLA and PDLA fibers, we observed an extensive decrease in the crystalline melting temperature until day 21, implying rapid hydrolysis. After day 21, the 1D1L, 3D1L and 1D3L fibers had a similar crystallinity trend until day 70. The hydrolysis behavior for the 3D1L and 1D3L could be divided into three steps: the initial fast hydrolysis of the amorphous domains, followed by HC domains’ hydrolysis, and then slow hydrolysis of the remaining SC domains.

**Fig. 10:**
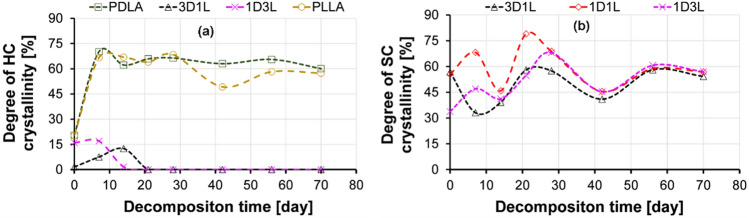
Change in the degree of crystallinity of **a** HC and **b** SC crystals over the hydrolytic decomposition time

The degree of crystallinity decreased after the first week, but the trends were not clear (Fig. 10, Table S 5 and Table S 6). In general, the water molecules’ access to chains inside the rigid crystalline domains was highly restricted. Hence, the increased degree of crystallinity at an early hydrolytic decomposition is mainly related to the degradation of amorphous domains.

The phenomenon can be explained by a fast water diffusion followed by hydrolysis of the molecular chains in the amorphous region. The 1D1L, 3D1L and 1D3L fibers exhibited a way lower change in the degree of crystallinity in the early decomposition times, indicating high hydrolysis resistance of the molecular chains in the amorphous domains. Results implied that SC amorphous domains also had somewhat higher water absorption resistance.

This phenomenon yielded a selective hydrolytic cleavage of chains in the amorphous regions of the molecular chains [56]. Therefore, hydrolysis occurred in water-soluble oligomers and monomers, while only some residual crystalline regions participated in the decomposition. The significant jump in the degree of crystallinity of the PLLA and PDLA fiber mat is related to the fast and extensive hydrolysis of the amorphous domains at the early decomposition times. Later, slowly degraded HC domains and related degradation products resulted in a slight decrease in the degree of crystallinity. The degree of crystallinity could decrease if hydrolysis is extended through the crystalline domains, which happens after the hydrolysis of the amorphous region has proceeded [57]. The 1D1L, 3D1L and 1D3L fibers exhibited a severe disintegration sourced by surface erosion in the hydrolytic medium. These results imply hydrolysis proceeded mainly along the crystalline boundary while the amorphous domains decomposed for the 1D1L, 3D1L and 1D3L fiber mats.

**Fig. 11:**
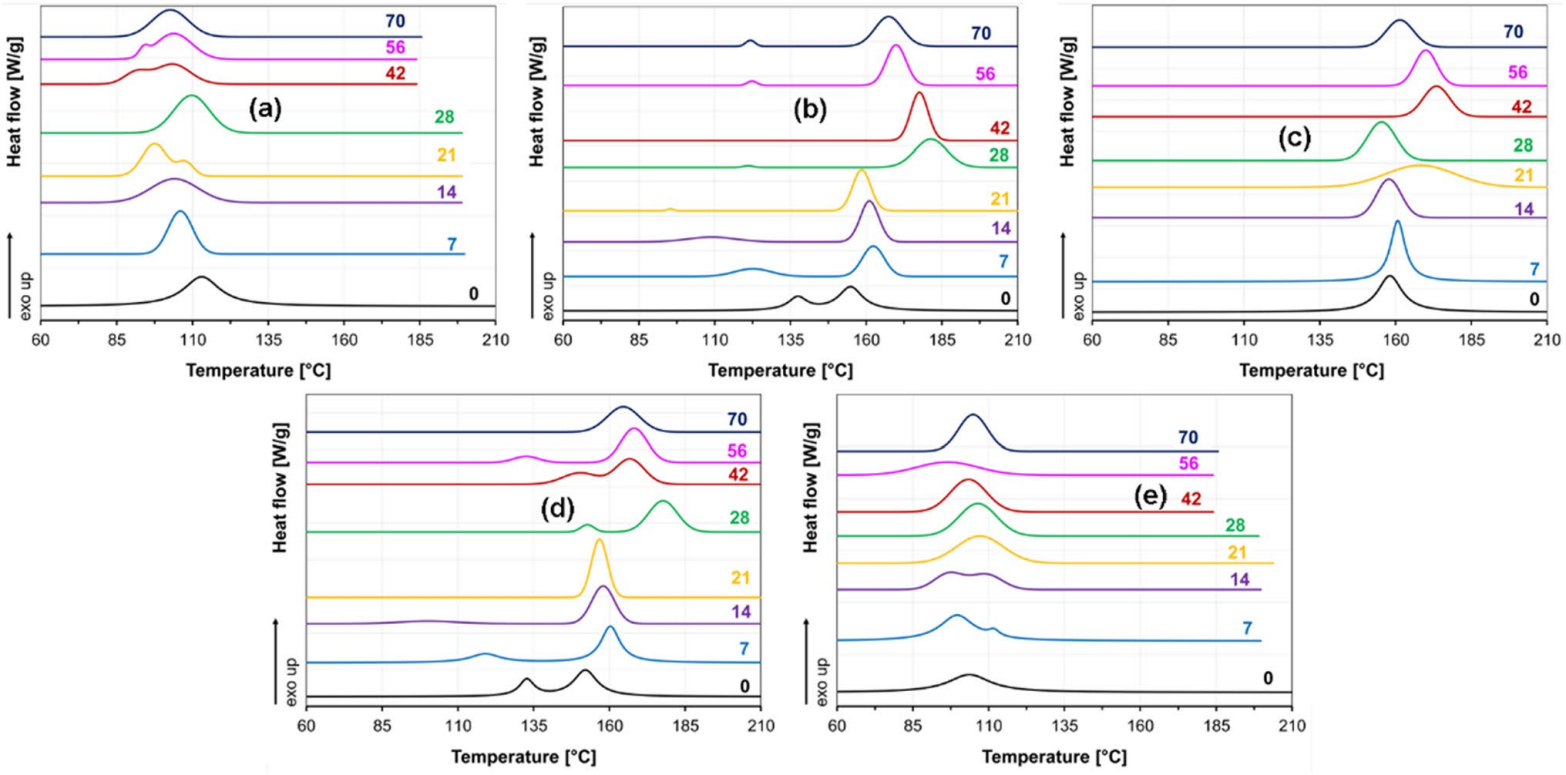
DSC cooling thermograms of the fiber mats; **a** PDLA, **b** 3D1L, **c** 1D1L, **d** 1D3L, and **e** PLLA at different decomposition times

DSC cooling cycles are shown in Fig. 11, while DSC data are given in Table S7 and Table S8. The 1D3L and 3D1L fibers had their exothermic HC and SC crystallization peaks until day 28. After that, both melting and crystallization peaks related to HC crystalline domains were disappeared. 3D1L and 1D3L fiber mats had double exothermic peaks from day 28, representing gradual hydrolytic decomposition behavior. Besides that, hydrolysis favors reducing the quantity of the amorphous domains that might increase chain mobility; thus, this allows chains to reorganize and crystallize, resulting in multiple peaks [22, 58]. The peak crystalline melting temperature of 3D1L and 1D3L materials deteriorated continuously after day 28. In contrast, low-temperature peaks related to HC domains (between 90 and 135 °C) exhibited lesser heat fusion and tended to disappear over time. Decreasing peak crystallization temperature might be related to shorter chains and initiation of the water into the crystalline domains [33, 56, 59]. On the other hand, PLLA and PDLA fibers had a continuous degradation, resulting in different crystalline species and hydrolytic decomposition time. The fully stereocomplex 1D1L fibers’ peak crystalline melting temperature decreased gradually only after day 42. These findings imply that the existence of both HC and SC crystal domains might also be favorable for the fast hydrolytic degradation of PLA fibers.

**Fig. 12:**
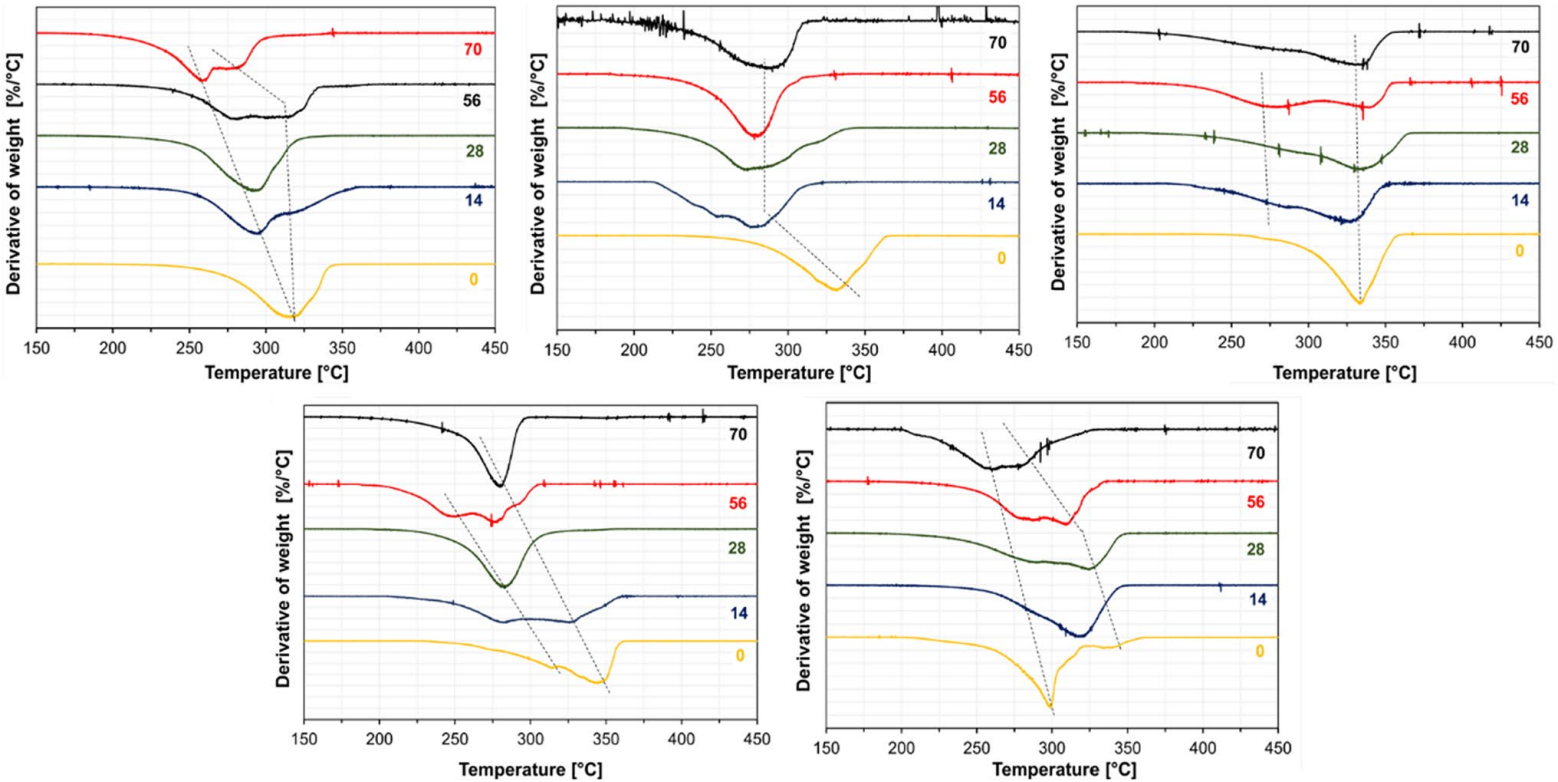
DTG curves of the fiber mats; **a** PDLA, **b** 3D1L, **c** 1D1L, **d** 1D3L, **e** PLLA at different decomposition times

In DTG analysis, all PLA fibers’ initial mass loss temperature and maximum mass loss rate temperature continuously shifted to lower temperatures over the decomposition time (Fig. 12).TGA data are given in Table S 9. DTG results are in line with the DSC findings. All PLA fiber mat’s DTG curves had shoulders or peaks at lower temperatures beside the main decomposition peak. That indicates that monomers or oligomers with increasing hydrolytic decomposition time are present [22, 33]. These are the product of chain scission and cleavage due to extensive hydrolysis. The lowest change in thermal decomposition temperature was obtained for the 1D1L fibers having SC crystals as crystalline species, followed by 3D1L. PLLA and PDLA fibers had the weakest thermal stability, and decomposition temperature shifted to lower levels over hydrolytic decomposition time.

**Fig. 13:**
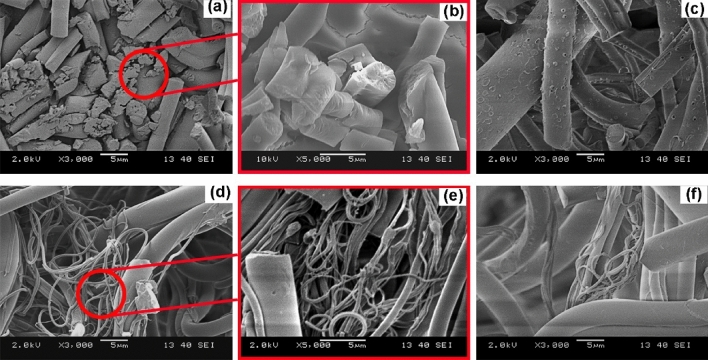
SEM images of the **a**–**b** PDLA, **c** 3D1L, **d**–**e** 1D1L and **f** 1D3L fibers hydrolytic decomposition at day 21

The SEM images demonstrating hydrolysis-induced structural decomposition in the fiber mat are shown in Fig. 13. The PLA fibers with HC showed similar degradation traces as at composting (Fig. 5c and f). The bulk erosion texture (Fig. 13 (a)-(b)) indicates the extensive and fast hydrolytic decomposition. Besides that, fibers with HC content disintegrated much faster than those containing single SC crystals. The morphology of 3D1L, 1D1L, and 1D3L samples exhibited mainly ruptures and surface erosion. In addition, SEM investigations implied that fiber diameter slightly increased due to water absorption (swelling).

**Fig. 14:**
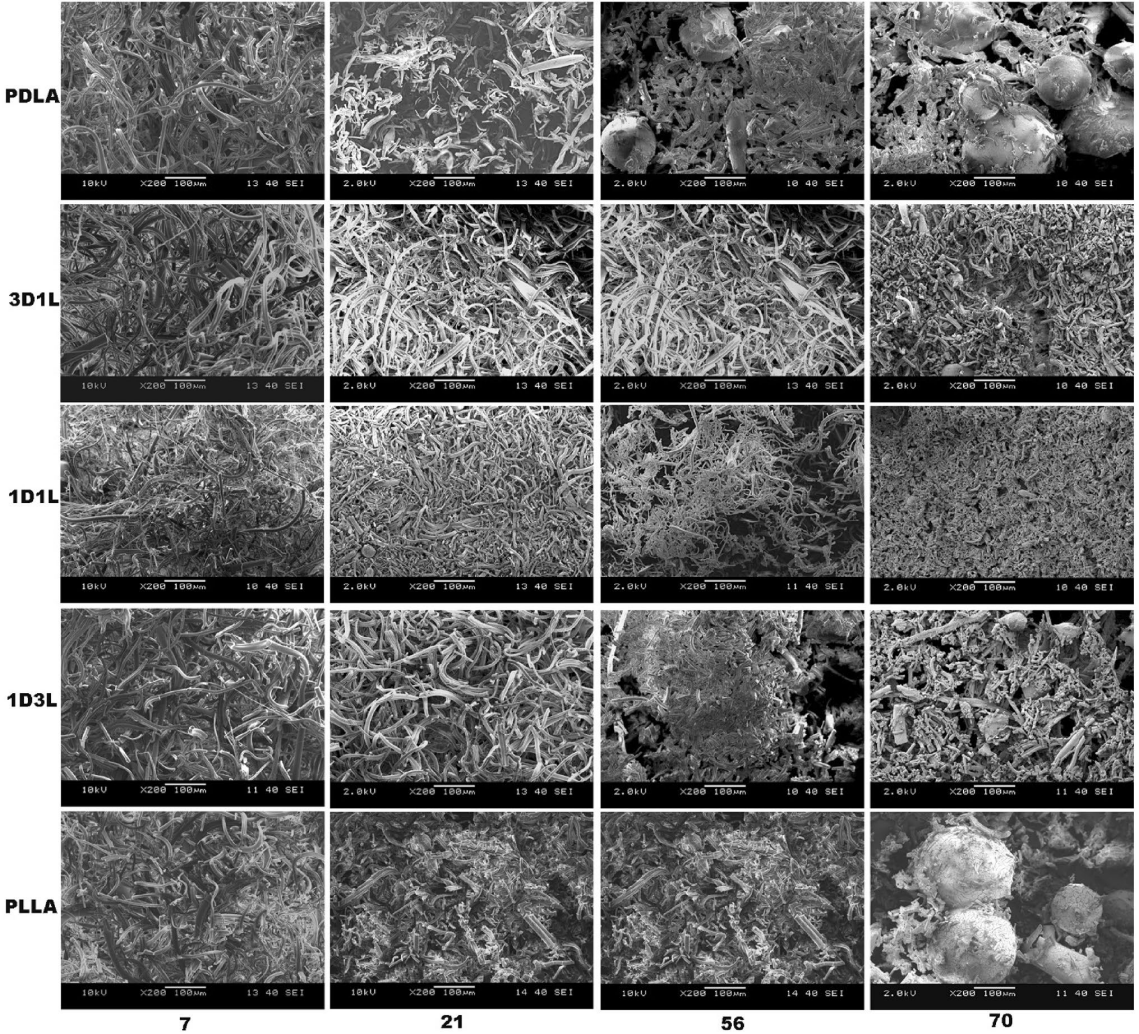
SEM images of MB fiber mats at different hydrolytic decomposition times

The SEM images (Fig. 14) also illustrate that hydrolysis resulted in severe disintegration of the PLLA and PDLA fiber mats. These first exhibited fiber ruptures, then short fibers accumulated and stuck to each other and formed small spheres, and they became a powder. After day 56, there is almost no trace of fiber shape. The stereocomplex 1D1L fibers more or less kept the fiber shape until day 70. On the other hand, 3D1L and 1D3L fibers suffered substantial fiber rupturing under hydrolysis. However, high PLLA content (1D3L fiber mat) showed fiber sticking and spheres of powders on the last stage of hydrolytic decomposition over 56 days, similar to PLLA and PDLA characteristics. It was also observed that all PLA fiber mats entirely disintegrated due to hydrolysis after 70 days in the hydrolytic medium. Results showed that the 1D1L fiber mat had high resistance to disintegration and decomposition. The PLLA fiber mat showed the lowest resistance to hydrolytic decomposition; the 1D3L fiber mat followed this. SEM studies revealed that the SC PLA fibers were more resistant to shape deterioration and erosion than PLLA and PDLA fibers in the hydrolytic medium.

## Conclusion

In this study, we produced PLA SC blends through melt mixing of commercially available PLLA and PDLA type PLA enantiomers. Subsequently, we generated PLA fine fiber mat via melt blowing. Findings showed that melt blowing led to the development of stereocomplex crystalline structure by shear-induced crystallization, which resulted in thinner fibers. The stereocomplex 1D1L fiber mat had an average fiber diameter of 1.8±0.3 μm, nearly 80% lower than PLLA and PDLA fibers, and it also had higher thermal stability. The SC (1D1L) fiber mat had nearly 60% and 30% higher specific strength than PLLA, PDLA, and asymmetric blends (e.g., 3D1L).

We then parallelly tested all PLA fiber mats’ soil burial composting and hydrolytic decomposition behavior in water. The PLA fiber mats’ entirely disintegrated in 40 days in the compost and 70 days in the water due to their high porosity and surface area. The DSC curves revealed that the crystal melting and crystallization peaks all shifted to lower temperatures, which is in line with the findings of the TGA measurements. We found that a high surface-to-volume ratio of fiber mats might boost a faster degradability in compost regardless of the molecular structure. SEM investigations showed that pure PLLA and PDLA fibers exhibited more severe and extensive surface to bulk erosion. On the other hand, the 3D1L, 1D1L and 1D3L blend fibers had mainly surface erosion, resulting in decreased amorphous content. The higher hydrolysis resistance and slower hydrolytic decomposition rate for these blends are related to inherently higher crystallinity, higher thermal and mechanical stability, and strong stereocomplex interactions.

We found that the degree of crystallinity and the crystalline structure dramatically influences the decomposition kinetics. The SC 1D1L fibers had the highest resistance against hydrolysis. The PDLA and PLLA fiber’s degree of crystallinity dramatically increased at the early stage due to the hydrolytic decomposition of the amorphous polymeric chains. On the other hand, PLA fiber with SC crystalline content exhibited high resistance to hydrolysis, for which we observed the degree of crystallinity changed slightly. Results showed that the SC PLA does not interfere with its aerobic and hydrolytic decomposition in the form of a fine fiber mat. Nonetheless, SC PLA enhances the fiber mat’s mechanical and thermal performance.

All in all, these results present a comprehensive understanding of the compost and hydrolytic decomposition behavior of PLA SC and HC fine fiber mats. These fiber mats could find broad applications because of their high porosity, high surface-to-volume ratio, self-bonding (e.g., no need for thermal bonding), moderate stiffness, lightweight and bio decomposition properties. The findings presented in this study will contribute to current efforts to generate green and sustainable fibers and related products for various industries and applications.

## Supplementary Information

Below is the link to the electronic supplementary material.

Supplementary material 1 (DOCX 1152.5 kb)
