# Supplementary material for: Decomposition Behavior of Stereocomplex PLA Melt-Blown Fine Fiber Mats in Water and in Compost
Source: J Polym Environ. 2022 Nov 28;31(4):1398–414. doi: 10.1007/s10924-022-02694-w (PMC9703430; doi:10.1007/s10924-022-02694-w)
Supplement: Supplementary file 1 — Supplementary material 1 (DOCX 1152.5 kb) [file 10924_2022_2694_MOESM1_ESM.docx]

**Supporting Information**

**Decomposition behavior of stereocomplex PLA melt-blown fine fiber mats in water and in compost**

Yahya Kara^1^ and Kolos Molnár^1,2^

1. Budapest University of Technology and Economics, Faculty of Mechanical Engineering, Department of Polymer Engineering, Műegyetem rkp. 3., H-1111 Budapest, Hungary

2. MTA–BME Research Group for Composite Science and Technology, Műegyetem rkp. 3., H-1111 Budapest, Hungary

Corresponding author: [molnar@pt.bme.hu](mailto:molnar@pt.bme.hu)


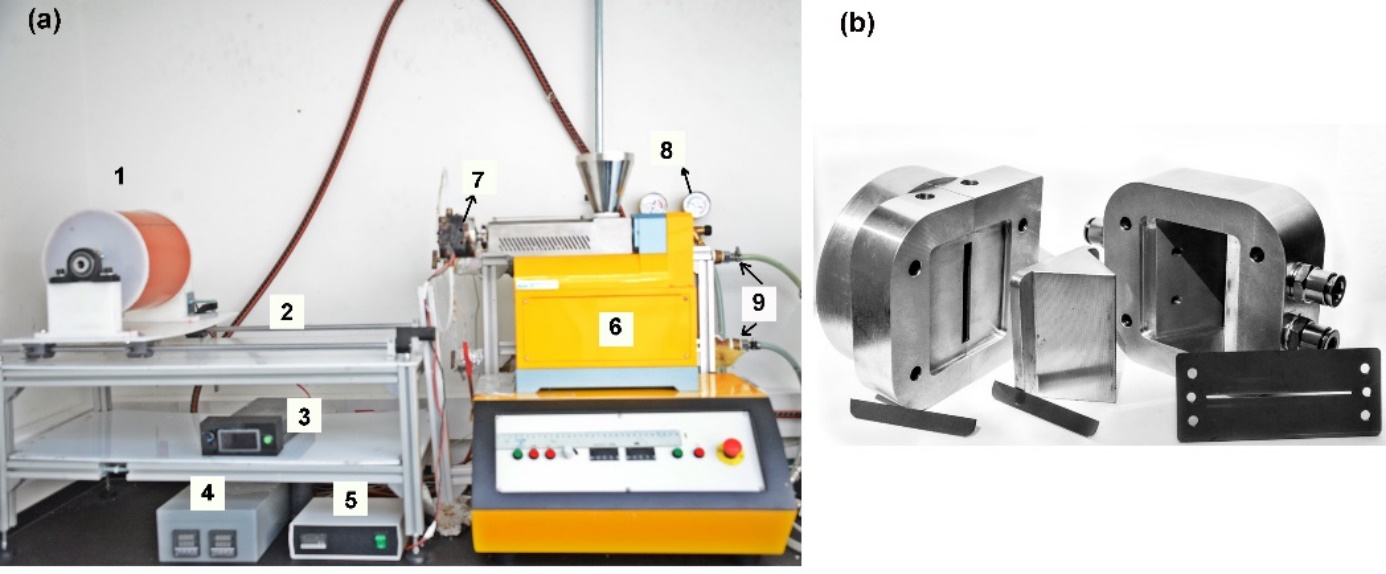


Figure S 1. Experimental melt blowing setup; (a) 1: collector drum, 2: linear guide for DCD adjustment, 3: collector motor controller, 4: air temperature controller, 5: die temperature controller, 6: extruder, 7: melt blowing die, 8: air pressure regulator, 9: air heaters; (b) melt blowing die parts


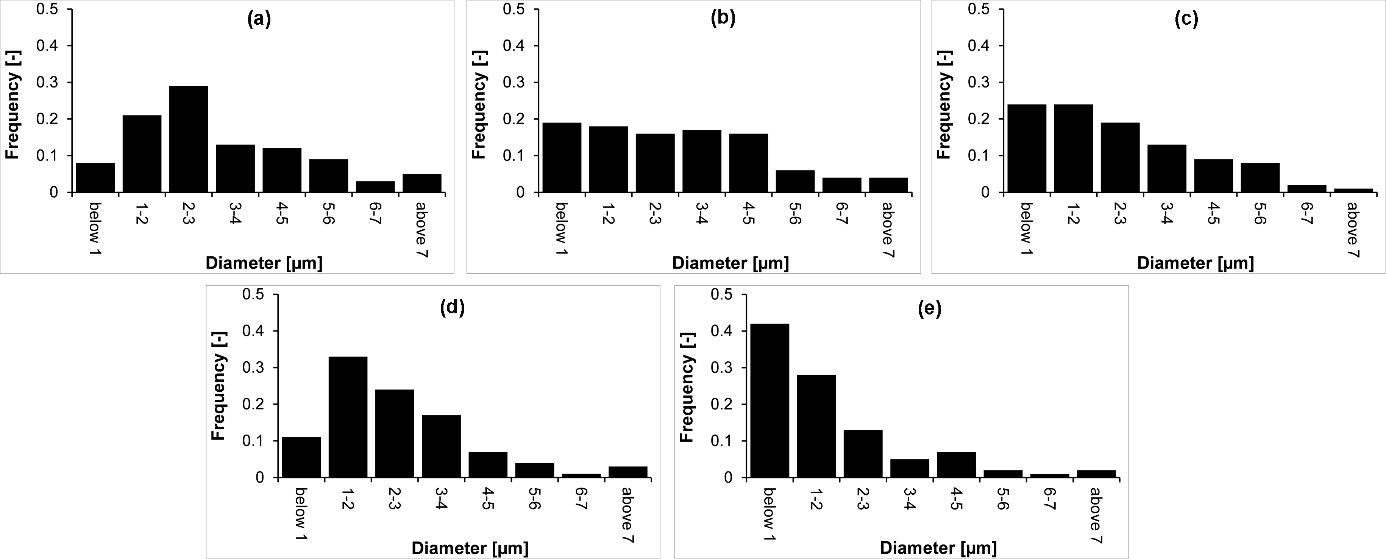


Figure S 2. MB fiber mats fiber diameter distributions (a) L, (b) D, (c) 3D1L, (d) 1D3L and (e) 1D1L


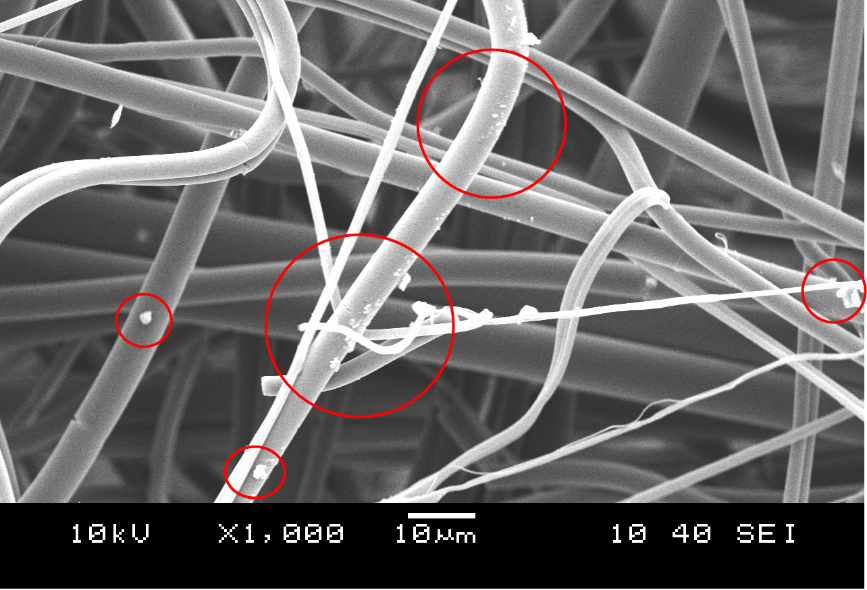


Figure S 3. SEM image of the 3L1D fiber mat defects


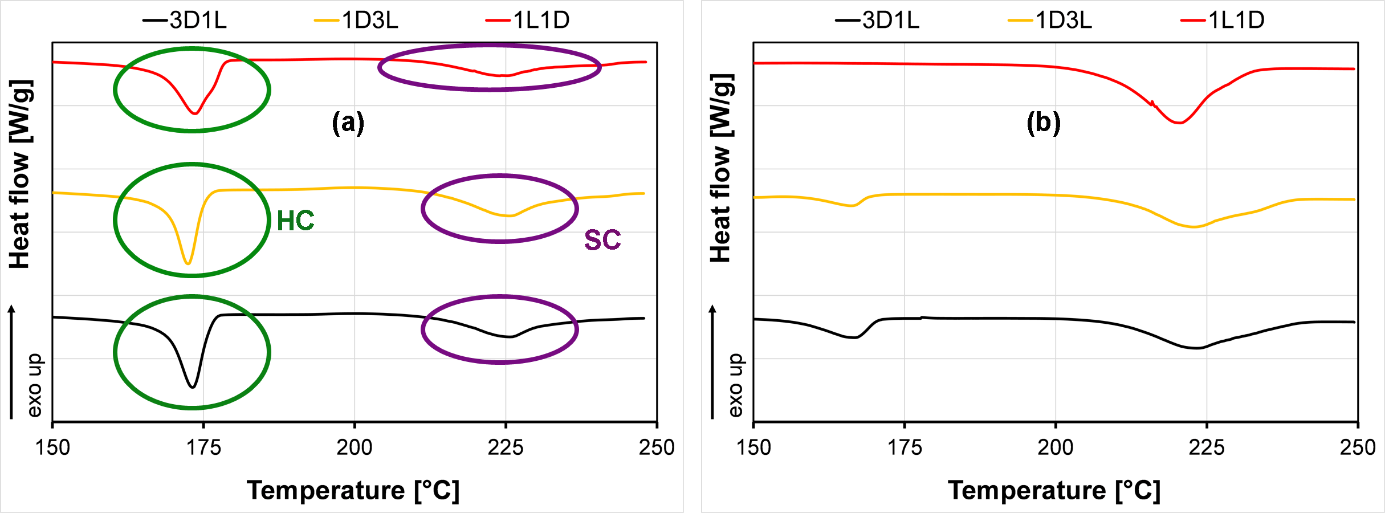


Figure S 4. DSC thermograms of (a) extrusion blends and (b) melt-blown fiber mats

Table S 1. DSC 1^st^ heating peak crystalline melting temperatures of the MB PLA fibers at different decomposition times

|  | 0 day | 7 day | 14 day | 0 day | 7 day | 14 day |
| --- | --- | --- | --- | --- | --- | --- |
|  | HC peak crystalline melting temperature [°C] | | | SC peak crystalline melting temperature [°C] | | |
| PDLA | 169.3 | 156.2 | 146.6 | - | - | - |
| 3D1L | 163.6 | 153.2 | - | 224.3 | 208.1 | 191.9 |
| 1D1L | - | - | - | 219.7 | 212.2 | 193.1 |
| 1D3L | 166.1 | 151.7 | - | 224.1 | 205.5 | 187.7 |
| PLLA | 165.7 | 159.2 | 145.2 | - | - | - |

Table S 2. DSC 1^st^ heating degree of crystallinity data for HC and SC PLA crystals of the MB PLA fibers at different decomposition times

|  | 0 day | 7 day | 14 day | 0 day | 7 day | 14 day |
| --- | --- | --- | --- | --- | --- | --- |
|  | Degree of HC crystallinity [%] | | | Degree of SC crystallinity [%] | | |
| PDLA | 20.4 | 65.2 | 41.8 | - | - | - |
| 3D1L | 1.5 | 2.3 | - | 56.7 | 27.5 | 3.7 |
| 1D1L | - | - | - | 55.5 | 69.1 | 55.4 |
| 1D3L | 15.9 | 5.5 | - | 33.8 | 49.4 | 3.2 |
| PLLA | 20.2 | 70.5 | 35.1 | - | - | - |

Table S 3. DSC 1^st^ heating HC peak crystalline melting temperatures of the MB PLA fibers at different hydrolytic decomposition times

|  | 0 | 7 | 14 | 21 | 28 | 42 | 56 | 70 |
| --- | --- | --- | --- | --- | --- | --- | --- | --- |
|  | HC peak crystalline melting temperature [°C] | | | | | | | |
| D | 169.3 | 158.8, 164.0 | 155.9 | 152.2 | 149.0 | 144.3 | 141.7 | 138.7 |
| 3D1L | 163.6 | 160.5 | 144.0, 157.3 | - | - | - | - | - |
| 1D1L | - | - | - | - | - | - | - | - |
| 1D3L | 150.3 | 145.6, 157.8 | 135.7, 151.2 | - | - | - | - | - |
| L | 165.7 | 160.2 | 151.3 | 147.7 | 144.7 | 142.0 | 139.0 | 138.6 |

Table S 4. DSC 1^st^ heating SC peak crystalline melting temperatures of the MB PLA fibers at different hydrolytic decomposition times

|  | 0 | 7 | 14 | 21 | 28 | 42 | 56 | 70 |
| --- | --- | --- | --- | --- | --- | --- | --- | --- |
|  | SC peak crystalline melting temperature [°C] | | | | | | | |
| D | - | - | - | - | - | - | - | - |
| 3D1L | 223.0 | 218.8 | 211.9 | 204.0 | 196.2 | 193.3 | 188.3 | 186.6 |
| 1D1L | 220.6 | 217.3 | 209.9 | 200.3 | 195.4 | 193.8 | 186.6 | 184.4 |
| 1D3L | 222.6 | 208.1 | 210.7 | 200.2 | 195.4 | 188.7 | 187.2 | 185.1 |
| L | - | - | - | - | - | - | - | - |

Table S 5. Degree of HC crystallinity of the MB PLA fibers at different hydrolytic decomposition times

|  | 0 | 7 | 14 | 21 | 28 | 42 | 56 | 70 |
| --- | --- | --- | --- | --- | --- | --- | --- | --- |
|  | Degree of HC crystallinity [%] | | | | | | | |
| D | 20.4 | 70.1 | 62.2 | 65.9 | 66.4 | 63.0 | 65.5 | 60.1 |
| 3D1L | 1.5 | 7.5 | 12.5 | - | - | - | - | - |
| 1D1L | - | - | - | - | - | - | - | - |
| 1D3L | 15.9 | 17.0 | 1.6 | - | - | - | - | - |
| L | 20.2 | 66.6 | 66.9 | 64.0 | 68.4 | 49.2 | 58.2 | 57.3 |

Table S 6. Degree of SC crystallinity of the MB PLA fibers at different hydrolytic decomposition times

|  | 0 | 7 | 14 | 21 | 28 | 42 | 56 | 70 |
| --- | --- | --- | --- | --- | --- | --- | --- | --- |
|  | Degree of SC crystallinity [%] | | | | | | | |
| D | - | - | - | - | - | - | - | - |
| 3D1L | 56.7 | 33.1 | 39.2 | 58.0 | 57.5 | 41.0 | 58.1 | 54.2 |
| 1D1L | 55.5 | 68.2 | 45.9 | 79.0 | 68.8 | 45.5 | 58.8 | 56.8 |
| 1D3L | 33.8 | 47.0 | 41.2 | 54.8 | 68.1 | 45.3 | 60.7 | 57.0 |
| L | - | - | - | - | - | - | - | - |

Table S 7. HC crystallization temperature of the MB PLA fibers at different hydrolytic decomposition times

|  | 0 | 7 | 14 | 21 | 28 | 42 | 56 | 70 |
| --- | --- | --- | --- | --- | --- | --- | --- | --- |
|  | HC crystallization temperature [°C] | | | | | | | |
| D | 110.2 | 106.0 | 104.0 | 97.5, 107.7 | 109.7 | 91.3, 103.4 | 93.9, 103.8 | 102.7 |
| 3D1L | 137.3 | 119.1 | 107.5 | 95.4 | 120.3 | - | 121.6 | 121.0 |
| 1D1L | - | - | - | - | - | - | - | - |
| 1D3L | 132.9 | 122.6 | 100.2 | - | - | - | 132.6 | - |
| L | 103.6 | 99.6, 111.7 | 96.9, 109.2 | 107.1 | 106.5 | 103.4 | 96.4 | 104.9 |

Table S 8. SC crystallization temperature of the MB PLA fibers at different hydrolytic decomposition times

|  | 0 | 7 | 14 | 21 | 28 | 42 | 56 | 70 |
| --- | --- | --- | --- | --- | --- | --- | --- | --- |
|  | SC crystallization temperature [°C] | | | | | | | |
| D | - | - |  | - | - | - | - | - |
| 3D1L | 154.9 | 160.3 | 159.6 | 158.5 | 180.6 | 176.8 | 169.1 | 166.7 |
| 1D1L | 158.2 | 160.7 | 157.8 | 168.2 | 155.4 | 173.5 | 170.0 | 161.4 |
| 1D3L | 152.1 | 162.4 | 157.9 | 156.8 | 177.7 | 150.3, 166.8 | 168.2 | 164.6 |
| L | - | - | - | - | - | - | - | - |

Table S 9. TGA 5 and 50 wt% mass loss temperatures of the MB PLA fibers at different hydrolytic decomposition times

|  | **T_5%_ [°C]** | | | | | **T_50%_ [°C]** | | | | |
| --- | --- | --- | --- | --- | --- | --- | --- | --- | --- | --- |
|  | **0** | **14** | **28** | **56** | **70** | **0** | **14** | **28** | **56** | **70** |
| D | 260.4 | 251.6 | 237.1 | 237.3 | 212.8 | 309.1 | 294.9 | 285.5 | 290.9 | 260.8 |
| 3D1L | 277.4 | 230.0 | 229.4 | 223.7 | 221.0 | 325.0 | 271.1 | 279.1 | 274.4 | 275.1 |
| 1D1L | 283.0 | 243.1 | 243.5 | 225.1 | 227.0 | 327.5 | 305.6 | 316.9 | 292.6 | 305.3 |
| 1D3L | 269.0 | 243.1 | 237.2 | 216.8 | 221.1 | 326.2 | 299.5 | 279.0 | 260.7 | 273.0 |
| L | 242.4 | 256.0 | 237.9 | 234.9 | 221.3 | 293.7 | 307.5 | 298.0 | 289.4 | 265.2 |
